# Correction: Hu et al. *Artemisia* Extracts Suppress H1N1 Influenza A Virus Infection by Targeting Viral HA/NA Proteins and Modulating the TLR4/MyD88/NF-κB Signaling Axis. *Pharmaceuticals* 2026, *19*, 275

**DOI:** 10.3390/ph19081170

**Published:** 2026-07-27

**Authors:** Zhongnan Hu, Hui Liu, Weihua Wu, Tayyab Ali, Adam Junka, Farukh S. Sharopov, Xuan Zou, Shisong Fang, Yanfang Sun

**Affiliations:** 1 College of Life Sciences and Medicine, Zhejiang Sci-Tech University, Hangzhou 310018, China; 2 Zhejiang International Joint Laboratory of Traditional Medicine and Big Health Products Development, Hangzhou 310018, China; 3 Shenzhen Center for Disease Control and Prevention, Shenzhen 518055, China; zzlh512@163.com (H.L.);; 4 “PUMA”, Platform for Unique Model Applications, Faculty of Pharmacy, Wroclaw Medical University, Borowska 211, 50-534 Wrocław, Poland; 5 Research Institution “Chinese-Tajik Innovation Center for Natural Products”, National Academy of Sciences of Tajikistan, Dushanbe 734063, Tajikistan

## Error in Figure

In the original publication [1], an accidental duplicate panel was found in Figure 9B during post-publication review. We have fully remade and replaced Figure 9B using raw data from independent repeated experiments. All revisions only involve image layout adjustment.

The authors state that the scientific conclusions are unaffected. This correction was approved by the Academic Editor. The original publication has also been updated.

**Figure 9:**
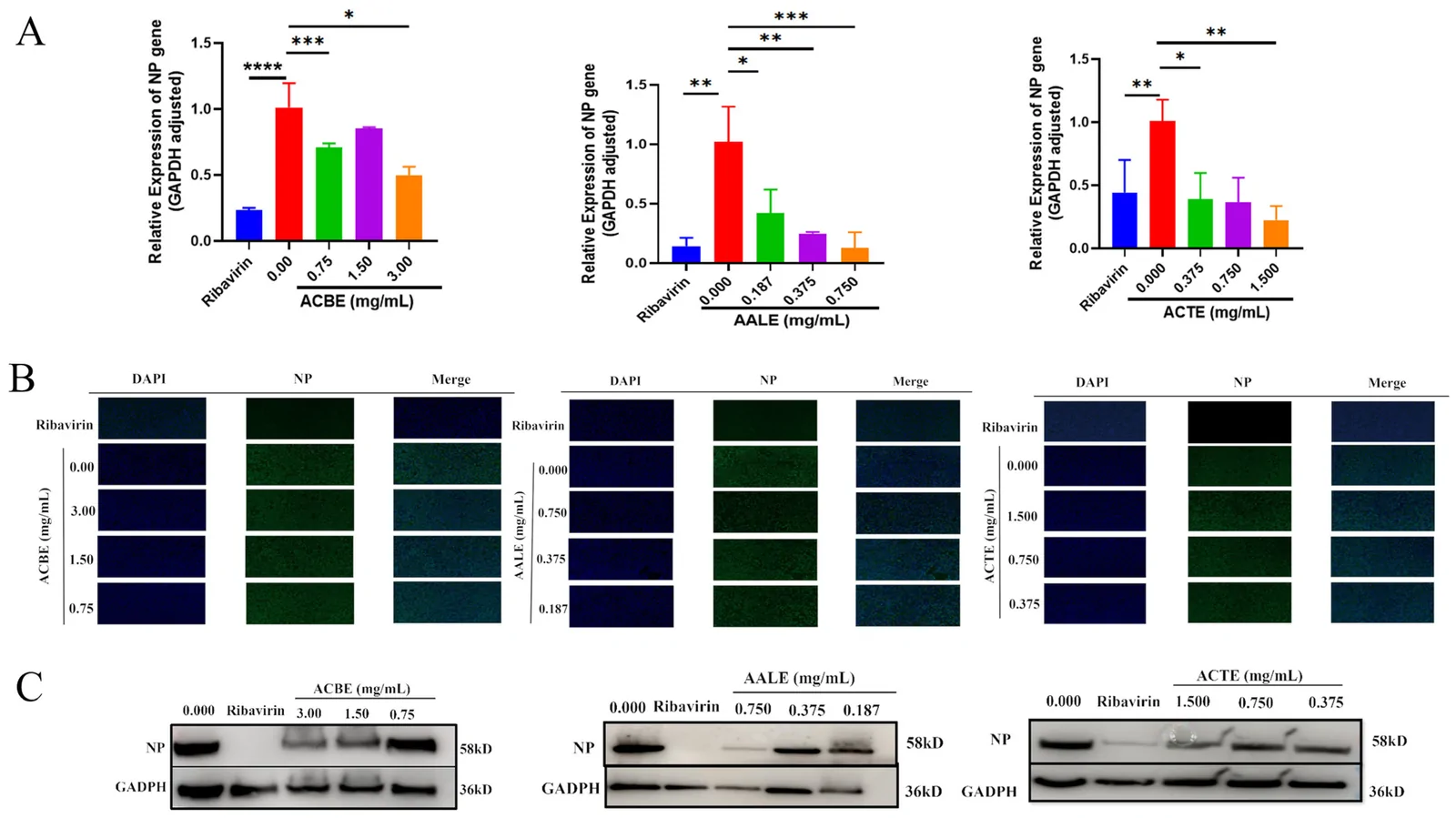
*Artemisia* L. plant extracts inhibit influenza virus infection through their therapeutic action. (**A**) The content of NP genes in each group, (**B**) the content of influenza virus NP protein in each group (Magnification is 10 × 10 (objective lens × eyepiece)), (**C**) the expression of NP protein in each group. Results are presented in the figure as the mean ± standard deviation (SD) of three independent experiments. One ANOVA with a *p*-value of less than 0.05 was considered statistically significant. * *p* < 0.05, ** *p* < 0.01, *** *p* < 0.001, **** *p* < 0.0001.
